# Non-primary progressive language impairment in neurodegenerative conditions: protocol for a scoping review

**DOI:** 10.1186/s13643-021-01589-6

**Published:** 2021-01-20

**Authors:** Sharon A. Savage, Aida Suárez-González, Alice Cassani, Ragaviveka Gopalan, Joshua Stott

**Affiliations:** 1 REACH: The Centre for Research in Ageing and Cognitive Health, College of Medicine and Health, University of Exeter, Exeter, UK; 2 School of Psychology, College of Engineering, Science and Environment, The University of Newcastle, Callaghan, New South Wales 2308 Australia; 3 Dementia Research Centre, UCL Queen Square Institute of Neurology, University College London, London, UK; 4 Discipline of Psychology, College of Life and Environmental Sciences, University of Exeter, Exeter, UK; 5 Research Department of Clinical, Educational and Health Psychology, Division of Psychology and Language Sciences, University College London, London, UK

**Keywords:** Aphasia, Dementia, Language intervention, Communication disorders

## Abstract

**Background:**

Progressive language difficulties arise in many neurodegenerative conditions, causing significant impact upon patients and families. This occurs most obviously in primary progressive aphasia (PPA) but can also occur within other forms of progressive disease. In these cases, language decline may be significant, but as they are not the presenting or dominant symptom, may be overlooked in favour of more prominent cognitive, behaviour or motor deficits. To date, there has been no systematic investigation into non-primary progressive aphasia. This scoping review aims to describe the currently reported language impairments found in non-language-led dementias and identify their clinical relevance, defined as the impact on everyday living. It also seeks to identify the reported interventions for language impairment in this patient group to-date.

**Method:**

We will conduct a scoping review of published studies that have assessed and/or treated aphasia in people diagnosed with a neurodegenerative condition other than primary progressive aphasia. The systematic search will include the electronic databases PubMed, MEDLINE, OVID-EMBASE, PsycINFO, and speechBITE, using search terms for specific non-language-led dementia subtypes. Findings will be mapped and described according to the type of language difficulties identified and rehabilitation approaches employed. Intervention studies will be evaluated for their methodological rigour using validated scales.

**Discussion:**

This scoping review will provide an overview of the types of aphasia found in neurodegenerative conditions where language dysfunction is not the primary focus. Current treatment approaches (and gaps in the provision of treatment) will be identified.

**Supplementary Information:**

The online version contains supplementary material available at 10.1186/s13643-021-01589-6.

## Background

The ability to express oneself and understand others through language is a central part of everyday life. When impaired, this can lead to disability and reduced quality of life [1]. Although memory impairments have typically been a point of focus when diagnosing and treating dementia (e.g., constituting an essential criteria in diagnostic classification systems such as ICD-10 until only recently), even early descriptions, such as those offered by Alois Alzheimer [2], include observations of language impairment within the context of neurodegenerative disease. In some cases, these impairments exceed those of other cognitive domains, a fact recognised by Marcel Mesulam, whose seminal work in the 1980s lead to the coining of the term ‘Primary Progressive Aphasia’ (PPA) [3, 4]. There are now three well described PPA syndromes [5] characterised by fluent speech with semantic breakdown, non-fluent speech with agrammatism and impaired repetition with phonologic errors. This recognition of PPA was an important step in raising awareness of non-memory-related disabilities associated with dementia, leading to the design of interventions that focused on improving expressive and receptive language abilities.

While non-primary in nature, progressive language difficulties have also been reported in many other neurodegenerative diseases. This includes reports in early-onset typical Alzheimer’s disease (AD) [6], Parkinson’s disease (PD) [7], dementia with Lewy bodies (DLB) [8], posterior cortical atrophy (PCA) [9], behaviour variant frontotemporal dementia (bv-FTD) and frontotemporal dementia with motor neurone disease (MND) [10], as well as the atypical parkinsonian syndromes of progressive supranuclear palsy (PSP) [11] and cortico-basal syndrome (CBS) [12]. Although clinically significant for patients and families, language symptoms in these conditions may be overlooked in favour of prominent non-language cognitive, behaviour or motor deficits. Thus, although such impairments may arise across a range of neurodegenerative syndromes, much is still to be learned regarding the nature and the prevalence of these language difficulties.

To date, systematic investigation of language deficits in these groups has been lacking, although it is likely that the language impairments which arise are variable in degree and severity, reflecting an overlapping continuum of profiles with the three classic primary progressive aphasia types. In recognising the commonalities between non-primary and primary progressive language impairments, there may arise the opportunity to use the knowledge gained from the treatment and support of people with PPA to assist those living with secondary language disorders.

We will therefore conduct a scoping review to map the literature concerning progressive language impairments in non-language-led neurodegenerative conditions (i.e. excluding PPA syndromes). In particular, we aim to provide an overview of the breadth and severity of language impairments, while also identifying current treatment approaches. In using a scoping review methodology rather than systematic review, we are able to combine information across various methodologies, including quantitative studies and case reports (common methodologies for research within this field), in a rigorous and systematic way.

## Methods/design

We will follow the PRISMA guideline (Preferred Reporting Items for Systematic Reviews and Meta-Analyses) extension for scoping review for conducting the review and preparing the report [13, 14]. See Additional file 1: PRISMA-ScR checklist.

### Objectives

This scoping review seeks to answer the following questions:

- What are the currently reported language impairments in non-language-led dementias?
- What is the clinical significance of these impairments? E.g. impact on quality of life, activities of daily living or any other measures used to evaluate these constructs.
- What are the reported language-based interventions for these patients?

Using the general framework outlined by Arksey and O’Malley [15] and guidance from the Joanna Briggs Institute Reviewer’s Manual [16], we will systematically scope the literature in non-language-led dementias, including early onset AD, PCA, PD dementia, DLB, behavioural variant FTD, FTD with MND, PSP and CBD. Literature relating to typical, late-onset Alzheimer’s disease or vascular dementia will be excluded. In the case of late-onset Alzheimer’s disease, the language profile is already well-characterised and known to include anomia and deterioration of semantic memory, without a well-defined aphasia emerging until very late into the disease [17]. This profile differs from the young onset and atypical forms of the disease, where focal pathology within the language network can arise earlier in the course of the disease and result in full aphasia syndromes. In vascular dementia, the different types of vascular pathology (e.g. diffuse small vessel disease vs repeated major cerebrovascular accidents, which are also variable in their size and location) result in non-uniform patterns of cognitive change. As a result, there is a lack of consistency within the clinical identity from which to collate evidence in this subtype [18].

### Information sources and search terms

The search will be conducted using the following databases: PubMed, MEDLINE, OVID-EMBASE, PsycINFO and speechBITE. The literature included will be indexed, published and peer-reviewed literature written in the English language with unlimited date range. Database-specific conventions and use of multiple search fields and filters will be customised for individual databases. English language filters will not be applied at this stage, with any resulting non-English articles removed manually during eligibility screening. In addition to the database searches, reference lists will be checked from key articles and reviews.

The following search terms will be used. These were developed in consultation with experienced researchers and librarians and were piloted:

(Early-onset Alzheimer’s disease OR early onset Alzheimer’s disease OR young-onset Alzheimer’s disease OR young onset Alzheimer’s disease OR Parkinson* dementia OR Parkinson* disease dementia OR dementia with Lewy bodies OR Lewy body dementia OR Posterior Cortical Atrophy OR frontotemporal dementia OR Pick’s disease OR Progressive Supranuclear Palsy OR Cortico-basal Syndrome OR cortico-basal degeneration OR corticobasal degeneration OR motor neurone disease OR FTD-MND OR MND-FTD OR ALS-FTD OR FTD-ALS) AND (language impairment OR communication disorder OR aphasia). See Additional file 2: Search Strategy for further details.

### Data management and screening

All results from the database searches will be exported into the Endnote software. Duplicate records will be removed. The screening of the candidate articles and the selection of qualified studies will then be conducted using a multi-level title-first method [19]. The primary reviewer (ASG) will independently inspect all the citations. Reviewers two and three (AC and RG) will then independently inspect 50% of the citations each, screening firstly by title, and then by abstract, to obtain an agreement regarding inclusion or exclusion of the article. Any differences will be discussed, and if required, resolved by consultation with an additional researcher (SS). At full text screening, a 10% sample of the articles selected by the primary reviewer will be screened by reviewers two and three to ensure the reliability of selection.

### Study selection

#### Population

Adults with a clinical diagnosis of early onset AD, PD dementia, DLB, PCA, PSP, CBS, bv-FTD or frontotemporal dementia in the context of motor neurone disease will be the focus of the review.

#### Concepts

Studies that assess and/or treat language difficulties in the target population will be included.

#### Context and comparators

Study selection will focus on articles published in English, where full text is available. Quantitative, mixed method, and case reports will be included. In some studies, comparison may be made between an intervention and a group who receive treatment as usual or no treatment. In other studies, this will be not applicable. Multiple reports that use data from the same study will be identified and collated, to avoid duplication of findings.

#### Exclusion criteria

- Studies focused on late onset AD, vascular dementia, mild cognitive impairment (MCI) or a non-neurodegenerative disease diagnosis
- Studies in which information regarding the nature of the language impairments is not provided
- Studies which do not assess or treat language difficulties, including studies which only assess non-linguistic or non-cognitive aspects of communication (i.e. motor speech, voice, prosody, dysarthria and/or oral apraxia)
- Treatments which are purely pharmacologically based or involve treatments such as electrical stimulation (transcranial magnetic stimulation or transcranial direct current stimulation).
- Articles where full text is not readily available or is not in English
- Studies where no original findings are presented (e.g. reviews/editorials/letters to editor)
- Non-peer reviewed material
- Conference abstracts
- Errata/correction of no significance to required data

Exclusion decisions and characteristics of excluded studies will be recorded. The search findings will be summarised using a PRISMA flow diagram [20].

### Data extraction and quality assessment

Information regarding type of language difficulties and treatment approaches taken will be extracted from each included article and recorded in a database. For studies describing the language impairments and impact upon everyday life, risk of bias will be evaluated using the *Standard Quality Assessment Criteria for Evaluating Primary Research Papers from a Variety of Fields* [21]. This method allows for simultaneous appraisal of both qualitative and qualitative dimensions of the same piece of work.

For quantitative studies reporting interventions, methodological rigour will be assessed using the PEDro-P scale [22] for group studies or the Risk of Bias in N-of-1 Trials [23] for single case designs, and where possible this information will be recorded directly from the speechBITE database (https://speechbite.com/rating-research-quality/).

### Collating, summarising and reporting results

Overall results will be tabulated according to the population studied, including diagnosis and severity of language difficulties, type of study and treatment approaches employed. A modified version of the charting form from the JBI template [16] (see Additional file 3: Charting form) will be used to extract data from each study. This has been tested on pilot searches and will be used to show the breadth and severity of language impairments, leading to a basic numerical summary. For intervention studies, summaries will also be tabulated according to level of evidence, with the highest quality designs presented first.

### Dissemination and ethics

The completed review will be written up and submitted for journal publication. Ethical approval is not required for this study.

## Discussion

This scoping review will provide an overview of the extent and nature of the research evidence about language deficits in people with non-language-led dementia subtypes, and will identify current treatment approaches. In doing so, there will be an opportunity to identify gaps in service delivery and evaluate the methodological rigour of current research in this area.

## Supplementary Information

**Additional file 1.** PRISMA-ScR checklist

**Additional file 2.** Search strategy

**Additional file 3.** Charting form

## Data Availability

Data sharing is not applicable to this article as no datasets will be generated or analysed during the current study.

## Abbreviations

AD: Alzheimer’s disease
ALS: Amyotrophic lateral sclerosis
bv-FTD: Behavioural variant frontotemporal dementia
CBS: Cortico-basal syndrome
DLB: Dementia with Lewy bodies
MCI: Mild cognitive impairment
MND: Motor neurone disease
PCA: Posterior cortical atrophy
PD: Parkinson’s disease
PPA: Primary progressive aphasia
PRISMA: Preferred Reporting Items for Systematic Reviews and Meta-Analyses.
PSP: Progressive supranuclear palsy
