# Supplementary material for: Non-primary progressive language impairment in neurodegenerative conditions: protocol for a scoping review
Source: Syst Rev. 2021 Jan 20;10:32. doi: 10.1186/s13643-021-01589-6 (PMC7816313; doi:10.1186/s13643-021-01589-6)
Supplement: Supplementary file 2 — Additional file 2. Search strategy [file 13643_2021_1589_MOESM2_ESM.docx]

**Additional file 2: Search strategy**

The proposed search strategy has been developed in consultation with the university librarian to ensure key articles are captured. The initial search will involve 5 databases: Pubmed, Medline, Ovid-Embase, PsycINFO and SpeechBITE. The resulting reference lists of identified articles will then be searched for additional studies.

An example of the search to be carried out in Pubmed is shown below.

**Pubmed search**

The search strategy involves entering search terms using the Pubmed default [All Fields] setting within the Advanced Search function, having cleared all default filters. Using the [All Fields] setting in Pubmed draws upon Medical Subject headings (MeSH) without limiting to specific qualifiers or subheadings.

1. (Early-onset Alzheimer’s disease OR early onset Alzheimer’s disease OR young-onset Alzheimer’s disease OR young onset Alzheimer’s disease)
2. (Parkinson* dementia OR Parkinson* disease dementia)
3. (dementia with Lewy bodies OR Lewy body dementia)
4. (Posterior Cortical Atrophy)
5. (frontotemporal dementia)
6. (Pick’s disease)
7. (Progressive Supranuclear Palsy)
8. (Cortico-basal Syndrome OR cortico-basal degeneration OR corticobasal degeneration)
9. (motor neurone disease OR FTD-MND OR MND-FTD OR ALS-FTD OR FTD-ALS)
10. 1 OR 2 OR 3 OR 4 OR 5 OR 6 OR 7 OR 8 OR 9
11. (language impairment OR communication disorder OR aphasia)
12. 10 AND 11

Resulting search details for Pubmed:

#1 Search: **Early-onset Alzheimer’s disease OR early onset Alzheimer’s disease OR young-onset Alzheimer’s disease OR young onset Alzheimer’s disease** Sort by: **Most Recent**

"alzheimer disease"[MeSH Terms] OR ("alzheimer"[All Fields] AND "disease"[All Fields]) OR "alzheimer disease"[All Fields] OR ("early"[All Fields] AND "onset"[All Fields] AND "alzheimer s"[All Fields] AND "disease"[All Fields]) OR "early onset alzheimer s disease"[All Fields] OR ("alzheimer disease"[MeSH Terms] OR ("alzheimer"[All Fields] AND "disease"[All Fields]) OR "alzheimer disease"[All Fields] OR ("early"[All Fields] AND "onset"[All Fields] AND "alzheimer s"[All Fields] AND "disease"[All Fields]) OR "early onset alzheimer s disease"[All Fields]) OR ("young-onset"[All Fields] AND ("alzheimer disease"[MeSH Terms] OR ("alzheimer"[All Fields] AND "disease"[All Fields]) OR "alzheimer disease"[All Fields] OR ("alzheimer s"[All Fields] AND "disease"[All Fields]) OR "alzheimer s disease"[All Fields])) OR (("young"[All Fields] OR "youngs"[All Fields]) AND ("age of onset"[MeSH Terms] OR ("age"[All Fields] AND "onset"[All Fields]) OR "age of onset"[All Fields] OR "onset"[All Fields] OR "onsets"[All Fields] OR "onsetting"[All Fields]) AND ("alzheimer disease"[MeSH Terms] OR ("alzheimer"[All Fields] AND "disease"[All Fields]) OR "alzheimer disease"[All Fields] OR ("alzheimer s"[All Fields] AND "disease"[All Fields]) OR "alzheimer s disease"[All Fields]))

**Translations**

**Early-onset Alzheimer's disease:** "alzheimer disease"[MeSH Terms] OR ("alzheimer"[All Fields] AND "disease"[All Fields]) OR "alzheimer disease"[All Fields] OR ("early"[All Fields] AND "onset"[All Fields] AND "alzheimer's"[All Fields] AND "disease"[All Fields]) OR "early onset alzheimer's disease"[All Fields]

**early onset Alzheimer's disease:** "alzheimer disease"[MeSH Terms] OR ("alzheimer"[All Fields] AND "disease"[All Fields]) OR "alzheimer disease"[All Fields] OR ("early"[All Fields] AND "onset"[All Fields] AND "alzheimer's"[All Fields] AND "disease"[All Fields]) OR "early onset alzheimer's disease"[All Fields]

**Alzheimer's disease:** "alzheimer disease"[MeSH Terms] OR ("alzheimer"[All Fields] AND "disease"[All Fields]) OR "alzheimer disease"[All Fields] OR ("alzheimer's"[All Fields] AND "disease"[All Fields]) OR "alzheimer's disease"[All Fields]

**young:** "young"[All Fields] OR "youngs"[All Fields]

**onset:** "age of onset"[MeSH Terms] OR ("age"[All Fields] AND "onset"[All Fields]) OR "age of onset"[All Fields] OR "onset"[All Fields] OR "onsets"[All Fields] OR "onsetting"[All Fields]

**Alzheimer's disease:** "alzheimer disease"[MeSH Terms] OR ("alzheimer"[All Fields] AND "disease"[All Fields]) OR "alzheimer disease"[All Fields] OR ("alzheimer's"[All Fields] AND "disease"[All Fields]) OR "alzheimer's disease"[All Fields]

#2 Search: **Parkinson* dementia OR Parkinson* disease dementia** Sort by: **Most Recent**

("parkinson*"[All Fields] AND ("dementia"[MeSH Terms] OR "dementia"[All Fields] OR "dementias"[All Fields] OR "dementia s"[All Fields])) OR ("parkinson*"[All Fields] AND ("disease"[MeSH Terms] OR "disease"[All Fields] OR "diseases"[All Fields] OR "disease s"[All Fields] OR "diseased"[All Fields]) AND ("dementia"[MeSH Terms] OR "dementia"[All Fields] OR "dementias"[All Fields] OR "dementia s"[All Fields]))

**Translations**

**dementia:** "dementia"[MeSH Terms] OR "dementia"[All Fields] OR "dementias"[All Fields] OR "dementia's"[All Fields]

**disease:** "disease"[MeSH Terms] OR "disease"[All Fields] OR "diseases"[All Fields] OR "disease's"[All Fields] OR "diseased"[All Fields]

**dementia:** "dementia"[MeSH Terms] OR "dementia"[All Fields] OR "dementias"[All Fields] OR "dementia's"[All Fields]

#3 Search: **dementia with Lewy bodies OR Lewy body dementia** Sort by: **Most Recent**

"lewy body disease"[MeSH Terms] OR ("lewy"[All Fields] AND "body"[All Fields] AND "disease"[All Fields]) OR "lewy body disease"[All Fields] OR ("dementia"[All Fields] AND "lewy"[All Fields] AND "bodies"[All Fields]) OR "dementia with lewy bodies"[All Fields] OR ("lewy body disease"[MeSH Terms] OR ("lewy"[All Fields] AND "body"[All Fields] AND "disease"[All Fields]) OR "lewy body disease"[All Fields] OR ("lewy"[All Fields] AND "body"[All Fields] AND "dementia"[All Fields]) OR "lewy body dementia"[All Fields])

**Translations**

**dementia with Lewy bodies:** "lewy body disease"[MeSH Terms] OR ("lewy"[All Fields] AND "body"[All Fields] AND "disease"[All Fields]) OR "lewy body disease"[All Fields] OR ("dementia"[All Fields] AND "lewy"[All Fields] AND "bodies"[All Fields]) OR "dementia with lewy bodies"[All Fields]

**Lewy body dementia:** "lewy body disease"[MeSH Terms] OR ("lewy"[All Fields] AND "body"[All Fields] AND "disease"[All Fields]) OR "lewy body disease"[All Fields] OR ("lewy"[All Fields] AND "body"[All Fields] AND "dementia"[All Fields]) OR "lewy body dementia"[All Fields]

#4 Search: **Posterior Cortical Atrophy** Sort by: **Most Recent**

("posterior"[All Fields] OR "posteriors"[All Fields]) AND ("corticalization"[All Fields] OR "corticalized"[All Fields] OR "cortically"[All Fields] OR "corticals"[All Fields] OR "cortices"[All Fields] OR "kidney cortex"[MeSH Terms] OR ("kidney"[All Fields] AND "cortex"[All Fields]) OR "kidney cortex"[All Fields] OR "cortical"[All Fields] OR "cerebral cortex"[MeSH Terms] OR ("cerebral"[All Fields] AND "cortex"[All Fields]) OR "cerebral cortex"[All Fields] OR "adrenal cortex"[MeSH Terms] OR ("adrenal"[All Fields] AND "cortex"[All Fields]) OR "adrenal cortex"[All Fields]) AND ("atrophie"[All Fields] OR "atrophy"[MeSH Terms] OR "atrophy"[All Fields] OR "atrophied"[All Fields] OR "atrophies"[All Fields] OR "atrophying"[All Fields])

**Translations**

**Posterior:** "posterior"[All Fields] OR "posteriors"[All Fields]

**Cortical:** "corticalization"[All Fields] OR "corticalized"[All Fields] OR "cortically"[All Fields] OR "corticals"[All Fields] OR "cortices"[All Fields] OR "kidney cortex"[MeSH Terms] OR ("kidney"[All Fields] AND "cortex"[All Fields]) OR "kidney cortex"[All Fields] OR "cortical"[All Fields] OR "cerebral cortex"[MeSH Terms] OR ("cerebral"[All Fields] AND "cortex"[All Fields]) OR "cerebral cortex"[All Fields] OR "adrenal cortex"[MeSH Terms] OR ("adrenal"[All Fields] AND "cortex"[All Fields]) OR "adrenal cortex"[All Fields]

**Atrophy:** "atrophie"[All Fields] OR "atrophy"[MeSH Terms] OR "atrophy"[All Fields] OR "atrophied"[All Fields] OR "atrophies"[All Fields] OR "atrophying"[All Fields]

#5 Search: **frontotemporal dementia** Sort by: **Most Recent**

"frontotemporal dementia"[MeSH Terms] OR ("frontotemporal"[All Fields] AND "dementia"[All Fields]) OR "frontotemporal dementia"[All Fields]

**Translations**

**frontotemporal dementia:** "frontotemporal dementia"[MeSH Terms] OR ("frontotemporal"[All Fields] AND "dementia"[All Fields]) OR "frontotemporal dementia"[All Fields]

#6 Search: **Pick’s disease** Sort by: **Most Recent**

"pick disease of the brain"[MeSH Terms] OR ("pick"[All Fields] AND "disease"[All Fields] AND "brain"[All Fields]) OR "pick disease of the brain"[All Fields] OR "pick s disease"[All Fields]

**Translations**

**Pick's disease:** "pick disease of the brain"[MeSH Terms] OR ("pick"[All Fields] AND "disease"[All Fields] AND "brain"[All Fields]) OR "pick disease of the brain"[All Fields] OR "pick s disease"[All Fields]

#7 Search: **Progressive Supranuclear Palsy** Sort by: **Most Recent**

"supranuclear palsy, progressive"[MeSH Terms] OR ("supranuclear"[All Fields] AND "palsy"[All Fields] AND "progressive"[All Fields]) OR "progressive supranuclear palsy"[All Fields] OR ("progressive"[All Fields] AND "supranuclear"[All Fields] AND "palsy"[All Fields])

**Translations**

**Progressive Supranuclear Palsy:** "supranuclear palsy, progressive"[MeSH Terms] OR ("supranuclear"[All Fields] AND "palsy"[All Fields] AND "progressive"[All Fields]) OR "progressive supranuclear palsy"[All Fields] OR ("progressive"[All Fields] AND "supranuclear"[All Fields] AND "palsy"[All Fields])

#8 Search: **Cortico-basal Syndrome OR cortico-basal degeneration OR corticobasal degeneration** Sort by: **Most Recent**

("cortico basal"[All Fields] AND ("syndrom"[All Fields] OR "syndromal"[All Fields] OR "syndromally"[All Fields] OR "syndrome"[MeSH Terms] OR "syndrome"[All Fields] OR "syndromes"[All Fields] OR "syndrome s"[All Fields] OR "syndromic"[All Fields] OR "syndroms"[All Fields])) OR ("cortico basal"[All Fields] AND ("degenerate"[All Fields] OR "degenerated"[All Fields] OR "degenerately"[All Fields] OR "degenerates"[All Fields] OR "degenerating"[All Fields] OR "degeneration"[All Fields] OR "degenerations"[All Fields])) OR ("corticobasal"[All Fields] AND ("degenerate"[All Fields] OR "degenerated"[All Fields] OR "degenerately"[All Fields] OR "degenerates"[All Fields] OR "degenerating"[All Fields] OR "degeneration"[All Fields] OR "degenerations"[All Fields]))

**Translations**

**Syndrome:** "syndrom"[All Fields] OR "syndromal"[All Fields] OR "syndromally"[All Fields] OR "syndrome"[MeSH Terms] OR "syndrome"[All Fields] OR "syndromes"[All Fields] OR "syndrome's"[All Fields] OR "syndromic"[All Fields] OR "syndroms"[All Fields]

**degeneration:** "degenerate"[All Fields] OR "degenerated"[All Fields] OR "degenerately"[All Fields] OR "degenerates"[All Fields] OR "degenerating"[All Fields] OR "degeneration"[All Fields] OR "degenerations"[All Fields]

**degeneration:** "degenerate"[All Fields] OR "degenerated"[All Fields] OR "degenerately"[All Fields] OR "degenerates"[All Fields] OR "degenerating"[All Fields] OR "degeneration"[All Fields] OR "degenerations"[All Fields]

#9 Search: **motor neurone disease OR FTD-MND OR MND-FTD OR ALS-FTD OR FTD-ALS** Sort by: **Most Recent**

"motor neuron disease"[MeSH Terms] OR ("motor"[All Fields] AND "neuron"[All Fields] AND "disease"[All Fields]) OR "motor neuron disease"[All Fields] OR ("motor"[All Fields] AND "neurone"[All Fields] AND "disease"[All Fields]) OR "motor neurone disease"[All Fields] OR "FTD-MND"[All Fields] OR "MND-FTD"[All Fields] OR "ALS-FTD"[All Fields] OR "FTD-ALS"[All Fields]

**Translations**

**motor neurone disease:** "motor neuron disease"[MeSH Terms] OR ("motor"[All Fields] AND "neuron"[All Fields] AND "disease"[All Fields]) OR "motor neuron disease"[All Fields] OR ("motor"[All Fields] AND "neurone"[All Fields] AND "disease"[All Fields]) OR "motor neurone disease"[All Fields]

#11 Search: **language impairment OR communication disorder OR aphasia** Sort by: **Most Recent**

"language disorders"[MeSH Terms] OR ("language"[All Fields] AND "disorders"[All Fields]) OR "language disorders"[All Fields] OR ("language"[All Fields] AND "impairment"[All Fields]) OR "language impairment"[All Fields] OR ("communication disorders"[MeSH Terms] OR ("communication"[All Fields] AND "disorders"[All Fields]) OR "communication disorders"[All Fields] OR ("communication"[All Fields] AND "disorder"[All Fields]) OR "communication disorder"[All Fields]) OR ("aphasia"[MeSH Terms] OR "aphasia"[All Fields] OR "aphasias"[All Fields] OR "aphasia s"[All Fields])

**Translations**

**language impairment:** "language disorders"[MeSH Terms] OR ("language"[All Fields] AND "disorders"[All Fields]) OR "language disorders"[All Fields] OR ("language"[All Fields] AND "impairment"[All Fields]) OR "language impairment"[All Fields]

**communication disorder:** "communication disorders"[MeSH Terms] OR ("communication"[All Fields] AND "disorders"[All Fields]) OR "communication disorders"[All Fields] OR ("communication"[All Fields] AND "disorder"[All Fields]) OR "communication disorder"[All Fields]

**aphasia:** "aphasia"[MeSH Terms] OR "aphasia"[All Fields] OR "aphasias"[All Fields] OR "aphasia's"[All Fields]
