# Supplementary material for: Non-primary progressive language impairment in neurodegenerative conditions: protocol for a scoping review
Source: Syst Rev. 2021 Jan 20;10:32. doi: 10.1186/s13643-021-01589-6 (PMC7816313; doi:10.1186/s13643-021-01589-6)
Supplement: Supplementary file 3 — Additional file 3. Charting form [file 13643_2021_1589_MOESM3_ESM.docx]

**Additional file 3: Charting form**

| **Scoping Review Details** | | |
| --- | --- | --- |
| Scoping review title | Non-primary progressive language impairment in neurodegenerative conditions. | |
| Research questions | 1. What are the currently reported language impairments in non-language led dementias? 2. What is the clinical significance of these impairments? E.g. impact on quality of life, activities of daily living or any other measures used to evaluate these constructs. 3. What are the reported language-based interventions for these patients? | |
| Objectives | - Systematically map the published research concerning progressive language impairments in non-language-led neurodegenerative conditions (i.e. excluding PPA syndromes). - Provide an overview of the breadth and severity of language impairment - Identify and summarise current treatment approaches | |
| **Evidence source Details and Characteristics** | | |
| Authors, year of publication | |  |
| Title, Journal | |  |
| Study design (e.g. case-control; cross-sectional) | |  |
| Participants (details e.g. sample size, dementia types included etc) | |  |
| **Details/Results extracted from source of evidence**(in relation to the concept of the scoping review) | | |
| Type of language impairment(s) | |  |
| Severity of language impairment(s) | |  |
| Associations between language impairment(s) and other areas of cognition | |  |
| Details of clinical significance of language impairment(s) (e.g. quality of life, ADLs) | |  |
| Interventions described | |  |
| SystQual score | |  |
| PEDRO-P score (for intervention studies) | |  |
